# multiplierz: an extensible API based desktop environment for proteomics data analysis

**DOI:** 10.1186/1471-2105-10-364

**Published:** 2009-10-29

**Authors:** Jignesh R Parikh, Manor Askenazi, Scott B Ficarro, Tanya Cashorali, James T Webber, Nathaniel C Blank, Yi Zhang, Jarrod A Marto

**Affiliations:** 1 Bioinformatics Program, Boston University, 24 Cummington Street, Boston, MA, 02115, USA; 2 Department of Cancer Biology and Blais Proteomics Center, Dana-Farber Cancer Institute, 44 Binney Street, Smith 1158A, Boston, MA, 02115-6084, USA; 3 Department of Biological Chemistry and Molecular Pharmacology, Harvard Medical School, 240 Longwood Avenue, Boston, MA, 02115, USA; 4 Department of Biological Chemistry, The Hebrew University of Jerusalem, Jerusalem, 91904, Israel

## Abstract

**Background:**

Efficient analysis of results from mass spectrometry-based proteomics experiments requires access to disparate data types, including native mass spectrometry files, output from algorithms that assign peptide sequence to MS/MS spectra, and annotation for proteins and pathways from various database sources. Moreover, proteomics technologies and experimental methods are not yet standardized; hence a high degree of flexibility is necessary for efficient support of high- and low-throughput data analytic tasks. Development of a desktop environment that is sufficiently robust for deployment in data analytic pipelines, and simultaneously supports customization for programmers and non-programmers alike, has proven to be a significant challenge.

**Results:**

We describe multiplierz, a flexible and open-source desktop environment for comprehensive proteomics data analysis. We use this framework to expose a prototype version of our recently proposed common API (mzAPI) designed for direct access to proprietary mass spectrometry files. In addition to routine data analytic tasks, multiplierz supports generation of information rich, portable spreadsheet-based reports. Moreover, multiplierz is designed around a "zero infrastructure" philosophy, meaning that it can be deployed by end users with little or no system administration support. Finally, access to multiplierz functionality is provided via high-level Python scripts, resulting in a fully extensible data analytic environment for rapid development of custom algorithms and deployment of high-throughput data pipelines.

**Conclusion:**

Collectively, mzAPI and multiplierz facilitate a wide range of data analysis tasks, spanning technology development to biological annotation, for mass spectrometry-based proteomics research.

## Background

Mass spectrometry-based proteomics, particularly liquid chromatography coupled to electrospray ionization, has become the predominant technique for identification and quantification of proteins in biological systems [1]. Growing demand for improved annotation of primary proteomics data with biological information from various public databases has catalyzed interest in the development of software tools to support integration of these data types. Unfortunately, a number of factors, including lack of experimental standardization, rapid introduction of novel mass spectrometry technology, and the evolution of proprietary file formats associated with proteomics platforms represent a significant hurdle to the development of efficient and comprehensive software frameworks.

To accommodate the emergent nature of proteomics-related technologies and the burgeoning number of databases that contain various biological annotations, data analytic systems must emphasize (i) intuitive and interactive interfaces, (ii) user-accessible coding frameworks to facilitate rapid prototyping of algorithms, and (iii) customizable sets of tools that can be readily integrated to provide pipelines that support a variety of proteomic workflows. Task specific Windows desktop applications such as MSQuant [2] and InsilicosViewer [3] can access a subset of native mass spectrometry data files directly and provide flexibility through adjustable parameters, but are not readily extended across the full spectrum of data analytic activities required in modern proteomics research. To address the full spectrum of analyses, open source projects such as The OpenMS Proteomics Pipeline (TOPP) [4] and ProteoWizard [5] offer a set of modular tools for generation of pipelines. The C++ coding environment of these tools is designed for performance and throughput, although researchers who lack programming experience often struggle to implement novel algorithms or other *ad hoc *tasks. Therefore, software libraries such as InSilicoSpectro [6] and mspire [7] have been developed based on high-level languages such as Perl and Ruby respectively. These libraries allow scripting of common data analysis tasks but cannot access raw binary data directly, and must rely instead on surrogate text files.

Historically the proprietary nature of binary files associated with proteomics technologies represented a significant obstacle to efforts aimed at development of integrated, desktop environments. One solution proposed specifically for mass spectrometry is extraction of native data to a common file format, typically a dialect of XML [8,9]. We [10] and others [11] have challenged the technical merits of this approach. Given that mass spectrometry manufacturers implicitly carry the burden of maintaining up-to-date libraries for access to their native data, we recently proposed that a common API [10] is a more rational solution for shared access to proprietary mass spectrometry files.

Here we define and implement a minimal API (mzAPI) that provides direct, programmatic interaction with binary raw files and we demonstrate that performance for practical tasks is significantly faster as compared to equivalent operations for access to mzXML files. We implement mzAPI in Python to maximize accessibility; similarly, mzAPI is exposed to users through multiplierz, a Python-based desktop environment that combines an intuitive interface with a powerful and flexible high-level scripting platform. Together, mzAPI and multiplierz support a wide range of data analytic tasks and facilitate rapid prototyping of novel algorithms. In addition, the multiplierz environment is designed with a "zero-infrastructure" philosophy, meaning that it can be deployed by end users who lack system administration experience or support. We demonstrate the capabilities of multiplierz through a variety of proteomics case studies such as (i) label-free quantitative comparison and interactive validation of datasets from multi-acquisition experiments, (ii) automatic quality control of mass spectrometer performance, (iii) improved peptide sequence assignment via deisotoping of MS/MS spectra, and (iv) assessment of phosphopeptide enrichment efficiency through programmatic fragment ion extraction.

## Implementation

### mzAPI: A Common API for Direct Access to Proprietary File Formats

As described above, direct access to native mass spectrometry data files is a key factor in the assembly of a powerful and flexible framework for proteomics data analysis. Towards this end, we define a minimal mzAPI as consisting of the following key procedures:

1. scan(time) → [(mz, intensity)]

2. scan_list(start_time, stop_time) → [(time, precursor)]

3. time_range() → (start_time, stop_time)

4. scan_time_from_scan_name (scan_name) → time

5. ric(start_time, stop_time, start_mz, stop_mz) → [(time, intensity)]

The first two procedures in mzAPI return: 1) individual scans in the form of a list of (mz, intensity) pairs, and 2) a catalog of all scan descriptions in the form of a list of (time, precursor) pairs in the experiment. In addition, the API provides: 3) 'time_range' that returns the earliest and latest acquisition times in the experiment, and 4) 'scan_time_from_scan_name' for translation of manufacturer-specific scan nomenclature to the mzAPI naming convention. We opted to rely on acquisition time as a common naming convention. In the case of LC-MS this is equivalent to chromatographic retention time. Finally, a fifth procedure generates a reconstructed ion chromatogram (RIC) for a given time and mass-to-charge range, returned as a set of (time, intensity) pairs. While in principle RICs can be generated using the first two calls, we believe that ubiquitous use of the RIC operation in proteomics data analysis justifies exposure of RIC extraction as a primitive in the API. Given that RIC extraction is provided by all manufacturer libraries, this procedure represents an excellent example of efficient re-use of native data system indexing and software.

We propose that a proprietary file format is considered mzAPI compliant when the manufacturer provides a freely available, and preferably redistributable, implementation of the aforementioned 5 core procedures, or an extended version that may evolve from a community-driven standardization effort. For example, ThermoFisher Scientific provides a data access library for .RAW files through the MSFileReader program, freely available for download at:  or . Naturally, additional procedure calls, such as charge state or signal-to-noise values for each isotope cluster in MS or MS/MS scans, can be incorporated into the mzAPI framework by essentially subclassing the core mzFile class.

As a basic test of file access speed, we compared the time required for random access of scans in an LC-MS acquisition from a ThermoFisher .RAW file using mzAPI (through its Python implementation) versus libraries provided by the manufacturer and included in the native Xcalibur file browser (note that mzXML data was not considered for this comparison since it does not provide access by acquisition time as a primitive in the RAP or RAMP API). Table 1 demonstrates that, as expected, random file access via mzAPI is slower than that obtained when working directly in the manufacturer's native environment. The performance of the common API could be further improved by implementation in C++ or C#, but we explicitly chose Python to maintain maximum flexibility through user defined scripts (see below). Interestingly, one consequence of the common API strategy is that it provides a direct measure of manufacturer data system efficiency, as evidenced by the additional time required for random access to scans in .WIFF versus .RAW files. Regardless of native file type, the use of a common API eliminates the need for storage and tracking of surrogate files; based on previous reports, this can be particularly problematic for full profile data files, which can grow significantly in size upon conversion to XML [9].

**Table 1:** Access Efficiency for Open and Proprietary Mass Spectrometry Data Files.

			**mean**	**sd**
**Random Access**		XDK (RAW)	1.81	0.01
(milliseconds)		mzAPI (RAW)	16.11	2.23
		mzAPI (WIFF)	216.10	8.58

**RIC Generation**	**GUI**	mzAPI (RAW)	0.58	0.03
(milliseconds)		Insilicos (mzXML)	12.17	1.93

	**Script**	mzAPI (RAW)	0.39	0.01
		InSilicoSpectro (mzXML)	15.68	0.06
		XCMS (mzXML)	37.46	4.74

Benchmarks for data access efficiency as determined for random scan access (orange fields) and generation of RICs (green fields). Three files were used: a RAW file acquired in data-dependent mode on an Orbitrap (ThermoFisher Scientific) with MS scans in profile mode and MS/MS scans in centroid mode; a WIFF file acquired in information dependent mode on a QSTAR Elite (MDS/Sciex) with all scans in profile mode; and a mzXML file generated using TPP (version 3.2) with the aforementioned RAW file as the source. For comparison of random access times a common single script was used to access both the RAW and WIFF file, by selecting 1000 scan times at random and that uniformly span the acquisition range. An equivalent process was implemented in C++ using the ThermoFisher XDK. For RIC generation in GUI mode (meaning that the resulting RIC is displayed by the software in an interactively accessible window) a script to animate the InsilicosViewer V1.5.1 was compared against a similar .mz script. For the non-GUI scenario we compared an R (version 1.12.1) script using the XCMS library as well as a Perl script using the InSilicoSpectro library (version 1.3.19) to another .mz script. The .mz scripts, R script, Perl script and C++ program are all available in Additional File 1. All measurements were repeated N = 20 times.

Given the multidimensional nature of mass-spectrometry data, extraction based on specific slices through the data space, rather than random file access, is a more relevant performance metric for mass spectrometry files. Generation of RICs is perhaps the best example of a data slice procedure supported by all manufacturer data systems. Consequently we next sought to test the performance of mzAPI for creation of RICs directly from a .RAW file. As a point of comparison we generated the corresponding mzXML file (using TPP version 4.0) [12] and extracted RICs using both a graphical user interface (GUI) based browser tool (InsilicosViewer version 1.5.1) [3] as well as the Perl-based InSilicoSpectro environment (version 1.3.19) [6] and the R-based XCMS (version 1.12.1), [13] scriptable interface platforms. Although the latter two are designed to access a number of third-party file formats, none of the GUI- or command-line based tools supports access to specified subsets (in chromatographic time) of the underlying data. As a result we generated RICs by extraction of a specific mass-to-charge range over the full data file, or in the case of InSilicoSpectro, which had no support for RIC generation, we simply timed the opening of mzXML files. While the mzXML schema includes a scan index that provides for random access to scans at speeds competitive with, or exceeding, proprietary data system (in this case ThermoFisher Xcalibur) [9], Table 1 shows that generation of specific data slices, or in this case RICs, is 5- to 10-fold faster when leveraging the underlying manufacturer's API compared to GUI or command line based access to mzXML (scripts used for all timings included in Additional File 1). This result supports the notion that pragmatic data access patterns are well supported by existing, albeit proprietary, manufacturer libraries, and more importantly, that these libraries can be efficiently utilized through a common and redistributable API.

### multiplierz: An Open-Source and Interactive Environment for Proteomics Data Analysis

**Figure 1:**
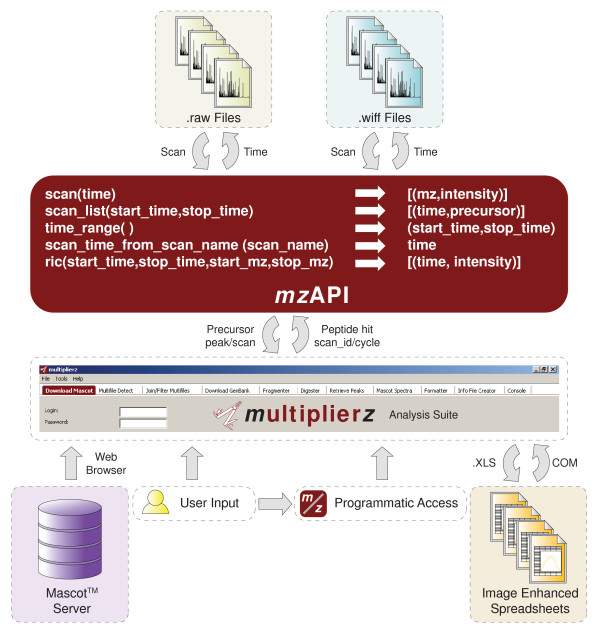
**A Common API and Desktop Environment for Mass Spectrometry Data Analysis**. The multiplierz environment provides a central point for user interaction with proprietary data files (via mzAPI), protein/peptide identification algorithms, publicly available annotation databases, and commercial reporting and spreadsheet tools. Our proposal calls for manufacturers to provide a minimal set of libraries for access to their native data files. *Ad hoc *data analysis tasks are supported through multiplierz scripting capability, including programmatic access for integration into data analytic pipelines.

We extend the functionality of mzAPI by integration into multiplierz, an open-source Python-based environment that provides a flexible framework for comprehensive analysis of proteomics data. Figure 1 illustrates our proposed implementation; all associated code and scripts are available for download at: . In the following sections, we provide a detailed description of core multiplierz capabilities.

#### Zero infrastructure integration of peptide identification and associated native mass spectrometry data

Regardless of final experimental goals, peptide identification is often the first or default operation performed subsequent to LC-MS data acquisition. We designed multiplierz to serve as a user-friendly, desktop tool for interaction with proteomics database search engines; consistent with our zero-infrastructure philosophy, X!Tandem [14] is fully integrated into the multiplierz installation package. Similarly we include support for automated retrieval of Mascot search results. In this case, the URL for a particular search is easily and unambiguously accessed using the Mascot job ID (after completion of the search, the Mascot ID is both on the search submission page and in the Mascot Daemon). The multiplierz module for downloading Mascot search results also allows input for Mascot-specific export options such as "Require Bold Red" and "Maximum Number of Protein hits." Multiple search results are specified using either a comma- or dash-separated list of Mascot Job IDs (e.g., 6556, 5878, 5120-5125). Users can optionally include Mascot MS/MS fragment annotation images (that are displayed in multiple Mascot report web pages) and embed them within a singular multiplierz report; thus multiplierz provides users with comprehensive Mascot information, including images, in a convenient and portable report (described below). Importantly, none of the above tasks require server level administrative privileges. For example, query of MS/MS peak annotations typically requires logon credentials within the web browser. multiplierz interacts with the browser to "screen scrape" MS/MS images and store them within the default report format. Users with full access to the Mascot server may parse results directly from the .DAT file using .mz scripts (multiplierz reports and .mz scripts and described below). Similar support is also provided for Protein Pilot [15] and OMSSA [16]. For maximum flexibility in conversion of parsed data from other search engines we include modules for generation of multiplierz-compatible spreadsheets.

Calculation of a false discovery rate (FDR) for peptide sequence identifications is one mechanism to assess the overall quality of search results [17,18]. multiplierz supports calculation of a FDR upon retrieval of peptide identification data from both forward and reverse database searches. The FDR for a given score threshold is calculated as the ratio of reverse database search identifications to that from the forward plus reverse searches, each with a score greater than or equal to the chosen threshold. The FDR thus represents the percentage of identified peptides in the forward search that would also be detected in the reverse database search. multiplierz identifies score thresholds for commonly used FDR (1%, 2%, and 5%) as well as calculates the FDR for each forward peptide score via an .mz Script (see below; scripts for generating a reverse database and calculating the FDR are included in Additional File 2).

Correlation of identified peptide sequences with specific features in the source mass spectrometry data, such as chromatographic peak width or maximum precursor intensity, is often complicated by the requirement for users to move between disparate programs and interfaces. The multiplierz desktop environment provides users with a centralized point of interaction with both search results and the underlying mass spectrometry files. For example, high-confidence peptide identifications may be used for direct generation of RICs across user-defined time and mass-to-charge ranges. Various metrics such as full peak width at half maximum (FWHM), peak area, and apex precursor intensity for peptide elution profiles are included in the output report. As described below these data are combined, annotated, and made available in portable, user-friendly reports.

#### Generation of portable multi-file reports

**Figure 2:**
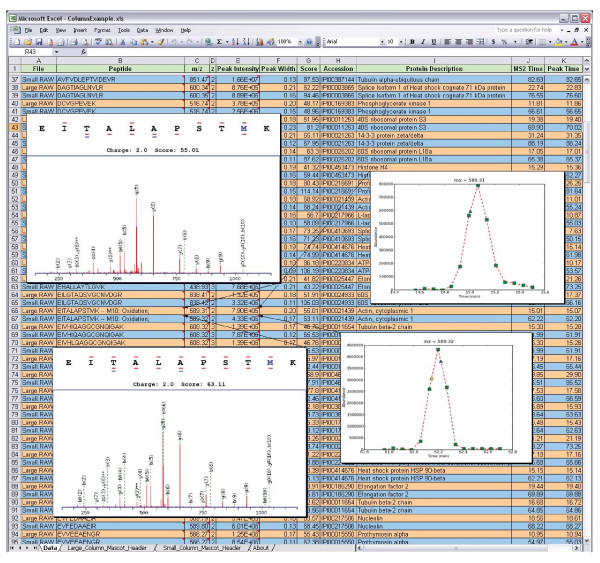
**Integration of Commercial Tools for multiplierz Reports**. A spreadsheet-based report from multiplierz analysis of 11 LC-MS analyses, designed to interrogate performance of LC column geometry and flow rate (see also Figure 5 and Additional File 5). For clarity the spreadsheet shows peptide entries and characterization data from the two extremes in column size and flow rate. Informative images are embedded within spreadsheet cell comments and are accessed by mouse-over, thus facilitating rapid visual inspection. Optional embedded images include: 1.) MS/MS spectra that are annotated with b- and y-type fragment ion labels, peptide sequence, search engine score (in this case Mascot peptide score), and precursor charge state. A color scheme highlights modified amino acids (in this case oxidized methionine) and those residues inferred from b- and y-type fragment ion assignments (horizontal lines, red and blue denote singly- and doubly-charged ions, respectively). 2.) RIC images in which MS scans are annotated with green squares, while yellow circles and blue triangles denote MS/MS scans for the precursor of interest, with the latter indicating the specific MS2 event described in the selected row of the spreadsheet. 3.) precursor region of the MS spectrum (not shown).

Consistent with our underlying motivation to combine open-source and commercial software where appropriate, we opted to export multiplierz results into Microsoft Excel. We take advantage of Excel's ability to store images in the worksheet (as comments), thereby creating an information-rich, yet portable, report that may be readily formatted to meet specific scientific journal data submission requirements [19]. Moreover, we note that the tendency towards analysis of increasingly complex mixtures along with continued efforts in relative protein quantification have placed increased emphasis on data reproducibility in proteomics experiments. Hence it has become common practice to derive a "proteomics result" from comparison, or other manipulation, of multiple mass spectrometry acquisitions. In support of this experimental paradigm, multiplierz includes "multi-detect" and "multi-filter" tools that provide users tremendous flexibility in filtering and collating (e.g., by common or unique proteins, peptides, post-translational modifications, charge state, etc.) each data file obtained from a multiple acquisition study. This feature provides database-like functionality without the need to install and maintain dedicated database servers. Importantly, all multiplierz functions provide spreadsheet-based output with optional embedded images (see Figure 2 and discussion below).

#### Interactive and dynamic analysis of native mass spectrometry data files

**Figure 3:**
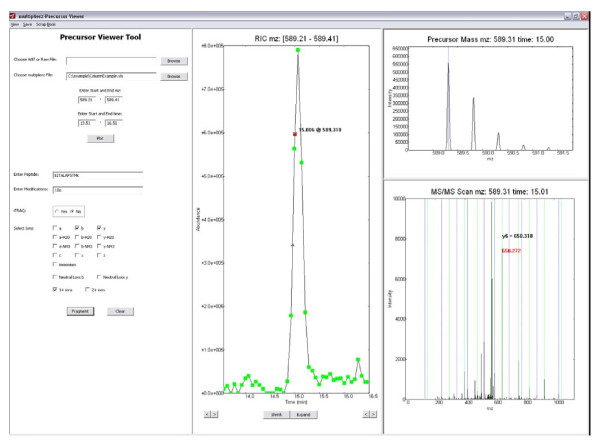
**Dynamic Visualization of Proprietary Mass Spectrometry Data Files**. The Peak Viewer tool in multiplierz provides interactive plots for precursor RICs, and corresponding MS and MS/MS scans, in centroid or profile modes. Green squares in the RIC denote MS scans and red triangles indicate MS/MS events. In addition, users may adjust the time or *m/z *range displayed in each data window. Verification of peptide sequence is facilitated by overlay of theoretical fragment ions on the MS/MS spectra. Users may dynamically evaluate multiple peptide assignment options by changing the proposed sequence or post-translational modification state in the left-most pane.

**Figure 4:**
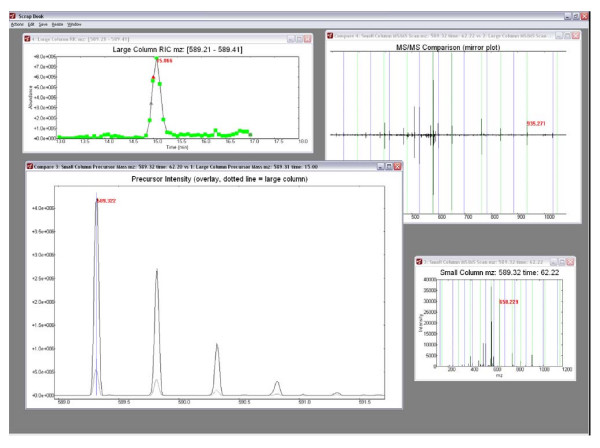
**Generation of Publication Quality Images**. A Scrapbook tool allows manipulation of Peak Viewer plot properties such as axes labels, titles, and size. Multi-plot comparison via the mirror and overlay functions provide further modes for in-depth, manual data interrogation. Users may export publication quality images from all Scrapbook plots.

The highly embedded, spreadsheet-based multiplierz reports provide a very flexible and user-friendly mechanism to query various metrics of the underlying native mass spectrometry data and quickly collate search results based on user-defined filter criteria. However, it is often the case that researchers must go beyond these general characterizations and focus on a small subset of their proteomics data in support of targeted biological questions. To enable this mode of data analysis, multiplierz includes a Peak Viewer tool (Figure 3) that provides dynamic and interactive plots for precursor RICs, and corresponding MS and MS/MS scans. Additionally, users can edit and export publication quality images through a built-in Scrapbook tool (Figure 4). Features of the Peak Viewer include (i) visualization of theoretical fragments superimposed on MS/MS spectra, (ii) automatic zoom-in display for iTRAQ and theoretical ions for rapid manual validation, and (iii) comparison of scans and RICs via mirror and overlay functions. For added convenience the Peak Viewer opens multiplierz spreadsheets and users can generate plots by a simple double-click on specific rows or peptide entries.

#### Other desktop tools

Researchers are increasingly focused on integration of disparate data types in order to better understand biological phenomena at the so-called network or systems level. As a first step in support of these and similar activities, multiplierz automatically downloads GenBank data over the internet based on an identified protein list, parses information such as gene ontology and domain classification, along with the corresponding Entrez Gene, HPRD, HGNC, and OMIM entries, and then creates hyperlinks directly in the spreadsheet reports. This and other tools including an *in silico *protein digestion tool and a peptide fragment calculator are described in Additional File 3.

#### Scripting capability for user-defined customization

While multiplierz includes many built-in features and tools, we also recognize the difficulty of building a "one size fits all" application given the diversity of ideas and efforts pursued within individual research laboratories. Hence multiplierz includes a command line console as well as scripting capability (through ".mz" scripts) which together support *ad hoc *data analysis tasks. The scripting capability is particularly useful for niche experiments or proteomics workflows not otherwise supported by other open-source or proprietary data systems. All multiplierz tools are available through both the desktop GUI as well as scriptable procedures. In addition, a pre-launch initialization ("rc.mz") script enables full customization of the application and its interfaces without recompiling the underlying code.

Finally, we note that programmatic access to mzAPI allows incorporation of multiplierz into automated data-analytic pipelines. For example, users can submit jobs through a laboratory information management system (LIMS). Upon completion of LC-MS acquisition(s) and database search(es), multiplierz executes .mz scripts to access both the search results and underlying .RAW or .WIFF file(s), in order to create a spreadsheet-based report. Users can be notified by email and access their results via the multiplierz desktop environment. Importantly, multiplierz spreadsheet reports, whether generated in low- or high-throughput mode, are portable and readily formatted in accordance with journal-specific requirements for proteomics data.

Collectively the features described above facilitate a wide range of data analysis tasks for mass spectrometry-based proteomics activities from technology development and evaluation to prioritization of protein identifications for subsequent biochemical validation. Importantly, multiplierz provides these capabilities to individual users at the desktop level.

## Results

In the following sections, we demonstrate the functionality of multiplierz through relevant examples based on data and results from work in our laboratory. Significantly we note that these examples encompass data generated on mass spectrometers manufactured by ThermoFisher Scientific and AB-SCIEX.

### Optimization of LC Assemblies and Methods

**Figure 5:**
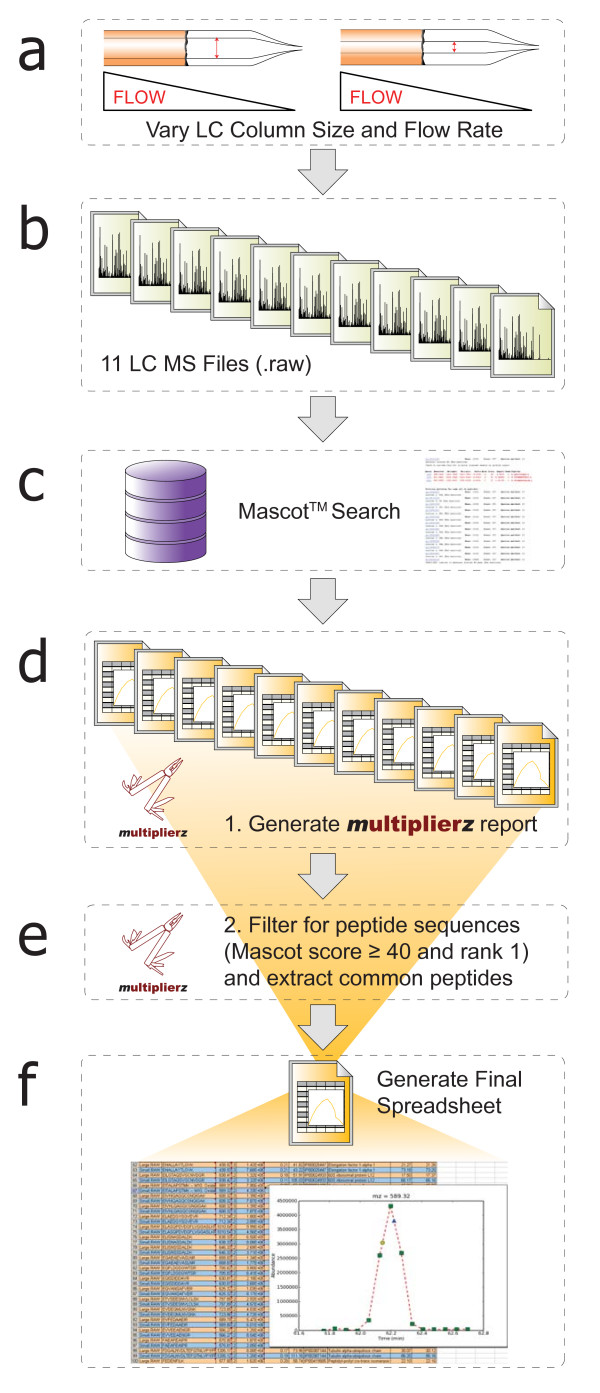
**Multiplierz -based Workflow for Analysis of LC Column Geometry and Flow Rate**. Relative performance improvement for analysis of peptides derived from whole cell lysate as a function of column size and flow rate (a). Original data contained ~90,000 MS/MS scans (b) encompassing almost 23,000 peptide assignments (combination of sequence, charge state, and modification), across 11 LC-MS acquisitions. In addition, multiplierz used Mascot-derived peptide identifications (c) to generate RICs, calculate full chromatographic peak width at half-maximum (FWHM), and determine precursor apex intensity (d). Common peptide sequences and associated analytical metrics are extracted (e) into a final, spreadsheet based report (f).

We recently described a novel protocol for fabrication of miniaturized LC-electrospray assemblies that provided significantly improved LC-MS performance [20]. Not surprisingly, elucidation of relevant analytical figures of merit required in-depth and large scale data analysis. Figure 5 shows the multiplierz-dependent workflow required to evaluate the relative performance improvement for analysis of tryptic peptides derived from whole cell lysate as a function of column size and flow rate (also see Additional File 5). From approximately 90,000 MS/MS scans encompassing almost 23,000 peptide assignments (combination of sequence, charge state, and modification) multiplierz identified 198 unique peptide sequences and modifications in common across 11 LC-MS acquisitions. In addition, multiplierz used Mascot-derived peptide identifications to generate RICs, calculate full chromatographic peak width at half-maximum (FWHM), and determine precursor apex intensity. The entire analysis was performed via the multiplierz GUI. Finally, the embedded RIC images facilitated rapid validation and comparison of chromatographic features.

Figure 2 (see above) shows an example of a multiplierz standard format report. To simplify the display we generated a comparison report (using multiplierz) for the two extremes in the 11 LC-MS acquisitions described above. The insets show examples of optional embedded images. We note that, unlike many web-based reports that often require frequent page updates, multiplierz images display immediately upon mouse-over, and hence facilitate rapid data validation and interrogation exercises.

### Optimization of Phosphopeptide Enrichment Methods

**Figure 6:**
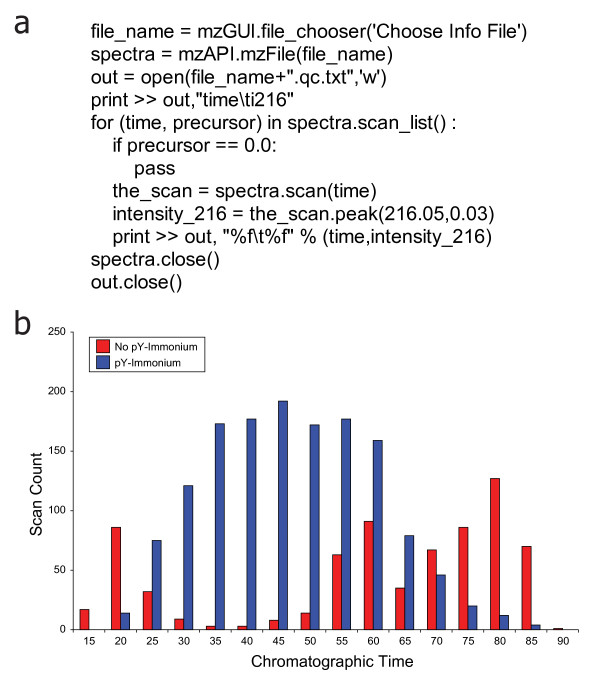
**User-defined Customization Through .mz Scripts**. A short .mz script (a) opens each MS/MS spectrum within a given LC-MS acquisition and returns those scans that contain the phosphotyrosine immonium ion (*m/z *= 216). The tab-delimited multiplierz output is readily opened in Excel and (b) a histogram view facilitates rapid evaluation of enrichment efficiency. In this example, the majority of peptides in the heart of the LC gradient (~25 - 65 min.) contain phosphotyrosine residues as evidenced by the presence of an *m/z *= 216 immonium ion.

In the aforementioned study, we leveraged the improved performance of our miniaturized LC-electrospray assemblies to elucidate signaling events in embryonic stem cells [20]. Our specific choice to focus on tyrosine phosphorylation as a direct probe of the molecular events required for self-renewal and differentiation in these cells required optimization of enrichment protocols for peptides carrying this rare post-translational modification. A typical strategy would be to simply adjust experimental conditions to yield a maximum number of phosphotyrosine sites subsequent to LC-MS/MS and database search. However, given the acutely low levels of tyrosine phosphorylation in embryonic stem cells, we chose instead to gauge enrichment efficiency based on the relative fraction of MS/MS scans that contained a phosphotyrosine immonium ion (*m/z *= 216.04) [21,22], irrespective of any putative peptide sequence assignment. This strategy allowed us to readily decouple low overall peptide yield from poor enrichment of phosphotyrosine containing peptides in experiments that generally provided modest numbers of peptide identifications (compared to typical large-scale proteomics studies). Figure 6a shows the .mz script used to probe MS/MS scans for the presence of a diagnostic fragment ion at *m/z *= 216. Note that Python's clear and concise syntax is readily accessible, as compared to that encountered with manufacturer libraries and data systems. Consistent with our reporting strategy, this script outputs a tab delimited file that we readily filter in Excel to generate a histogram view of our phosphotyrosine enrichment efficiency (Figure 6b).

**Figure 7:**
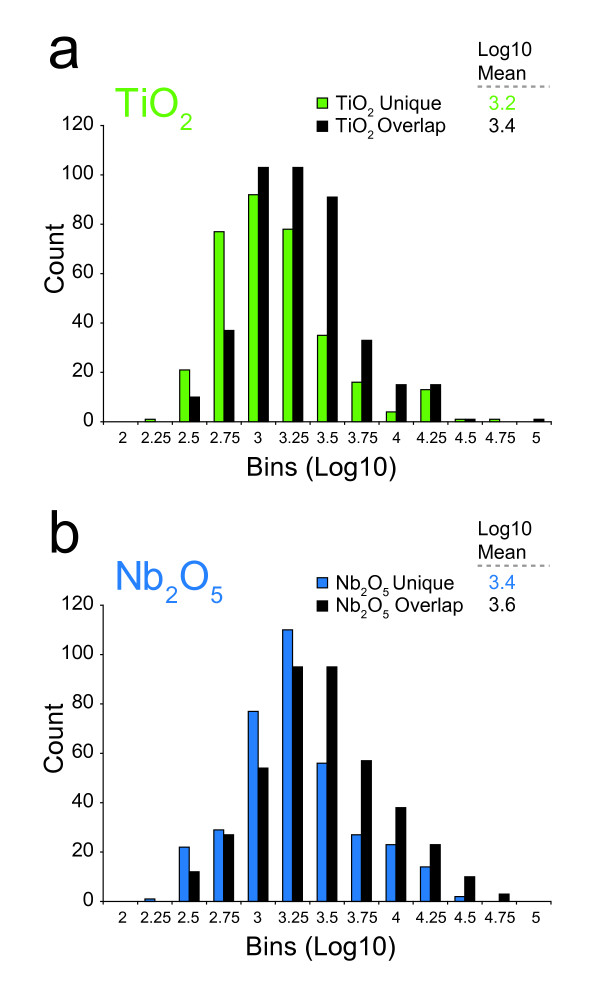
**Quantitative Comparison of Phosphopeptide Enrichment Methods**. Histogram distributions of peak heights for unique and overlapping phosphopeptides detected in conjunction with (a) (TiO2)-, and (b) (Nb2O5)-based enrichment. The intensity distributions for phosphopeptides assigned uniquely to either metal oxide did not differ significantly from the intensity distributions for commonly detected phosphopeptides, indicating that the unique precursors were not confined to low signal-to-noise regions. Reprinted from [23] by permission from the American Chemical Society.

In a separate report we described the novel application of niobium(V) oxide (Nb_2_O_5_) for global enrichment of phosphopeptides from complex, biologically derived mixtures [23]. The "multi-detect" and "multi-filter" tools in multiplierz were used to compare phosphopeptides enriched via Nb_2_O_5 _and TiO_2_, (the current standard), and detected across multiple LC-MS/MS analyses. Furthermore, to assess potential bias introduced by the stochastic nature of MS/MS, we compared the precursor peak intensities of unique versus commonly detected phosphopeptides that resulted from each method, and confirmed that Nb_2_O_5 _and TiO_2 _exhibited an empirically useful degree of divergence with respect to phosphopeptide enrichment (Figure 7, reprinted from Ficarro et al. [23] by permission from the American Chemical Society).

### Improved Peptide Sequence Assignment via De-isotoped MS/MS Spectra

In another recent study, we optimized performance of orbitrap HCD MS/MS through systematic exploration of various instrument and post-acquisition parameters [24]. In the context of this work, we observed that high charge state (z > 2+) precursors were frequently not assigned to a peptide sequence despite an otherwise high quality fragment ion spectrum. We speculated that the presence of multiple isotope peaks per fragment ion in the high resolution Orbitrap MS/MS scans may degrade the sensitivity of the search algorithm, resulting in fewer high-confidence sequence assignments. Therefore, we generated an .mz Script (see Additional File 2) that de-isotoped [25] each fragment ion cluster and output a charge state reduced peak list for submission to Mascot. A variety of parameters can be used to adjust the stringency of spectrum filtering such as maximum charge state, minimum fragment ion mass-to-charge ratio, as well as an option to remove any precursor signal that may remain in the MS/MS spectrum. Overall we realized an approximate 30% gain in the number of high-confidence (Mascot score > 30) peptide sequence assignments for high charge state precursors (Table 2).

**Table 2:** Improved Peptide Sequence Assignment via De-isotoped MS/MS Spectra.

**Experiment**	**# Peptides Before Deisotoping**	**# Peptides After Deisotoping**	**# New Peptides**	**% New Peptides**
1	218	271	53	31%

2	178	208	30	17%

3	204	250	46	33%

4	379	442	63	34%

5	320	391	71	35%

**Average**	**259.8**	**312.4**	**52.6**	**30%**

High resolution Orbitrap HCD MS/MS spectra of high charge state (z > 2+) precursors were frequently not assigned to a peptide sequence due to the presence of multiple isotope peaks per fragment ion. We processed the peak lists (MGF format) using an .mz script to only retain mono-isotopic and singly charged fragment ion peaks. Subsequent research of these data provided an average increase of 30% in the number of high-confidence (Mascot score > 30) peptide assignments across 5 LC-MS runs.

### Label-Free Quantitative Proteomics

**Figure 8:**
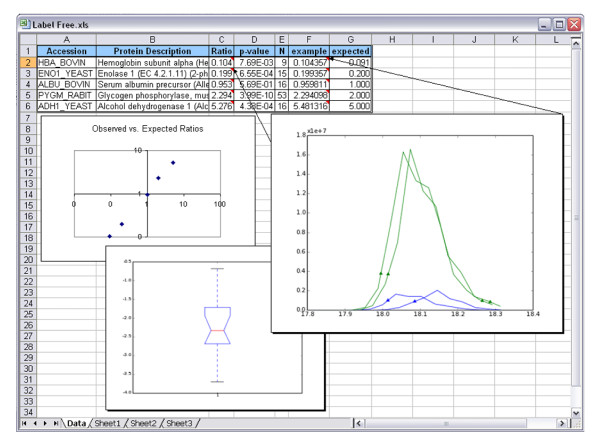
**Label-Free Relative Protein Quantification**. A multiplierz Excel report provides data analytic figures of merit, including: (a) the ratio of each protein across two conditions with an embedded box plot that illustrates the distribution of feature-level ratios, where each feature is defined as a (peptide, modification, charge state) combination; (b) p-value for the significance of the ratio; (c) the number (N) of features underlying the protein quantification; (d) the ratio and embedded RIC plot (showing all RICs used to quantify the peptide - colored by sample source) from the peptide most representative of the final protein ratio; (e) "expected" field is added manually by the user based on the experimental design. Users may also generate associated graphs using native plotting capabilities in Excel.

Relative protein quantitation can be achieved via a label-free approach whereby tryptic digests of protein samples are analyzed without incorporation of stable isotope labels; the resulting peak intensities (or areas) for the constituent peptides are combined and compared across samples as well as within replicates [26]. Typically 3-5 replicates of each sample are required to account for non-systematic errors associated with shifts in chromatographic elution time, temperature, electrospray stability, etc. For very large studies, performed across extended periods of time and multiple labs, complex software is typically required to combine, align and analyze the resulting native mass spectrometry files. In contrast, we demonstrate a strategy similar to the one described by Bondarenko et al. [27], which is deployed entirely within multiplierz: extraction of MS/MS peak lists, X!Tandem based peptide identification, and the generation of common sequences (detected in at least k out of the n RAW files being analyzed) are implemented directly from the multiplierz menu-system. Finally, feature extraction, quantitation, and report generation is performed via an additional mz script (see Additional File 2). Figure 8 shows an Excel-based report for label-free analysis of two standard protein mixtures (5 proteins each, containing ratios of 1:11, 1:5, 1:1, 2:1 and 5:1, respectively, and analyzed in duplicate).

### Automated Quality Control of Mass Spectrometer Instrument Performance

**Figure 9:**
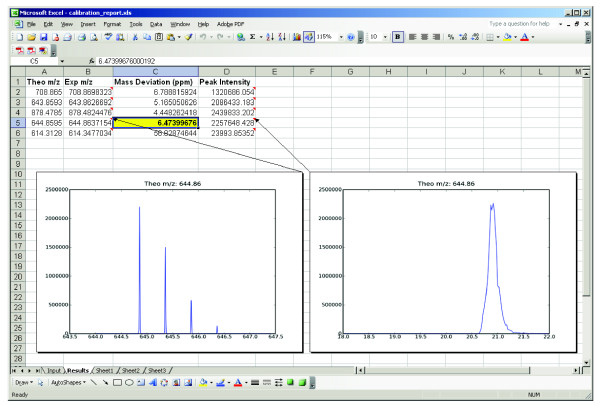
**Instrument Calibration Quality Control Report**. An .mz script is used to automatically generate a spreadsheet report that indicates mass errors (in ppm) for a set of standard peptides. Images of the precursor isotope distribution and reconstructed ion chromatogram are embedded within the report for rapid confirmation of mass spectrometry and chromatographic performance.

High throughput or other core-type operations designed to run in an unattended manner benefit from automated quality control assessment of platform performance. For example, periodic confirmation of measured peptide mass accuracy is required to ensure the integrity of instrument calibration routines. Towards this end, we created a short .mz script (see Additional File 2), which extracts measured mass-to-charge values for a list of standard peptides from a native data file, and automatically calculates mass errors. The output is a calibration report (Figure 9) that shows a reconstructed ion chromatogram and experimental mass accuracy for each standard peptide. The measured mass errors may be used as input for mass tolerance parameters in subsequent database search algorithms (e.g., Mascot, SEQUEST, X!Tandem, etc.) for peptide sequence identification.

**Figure 10:**
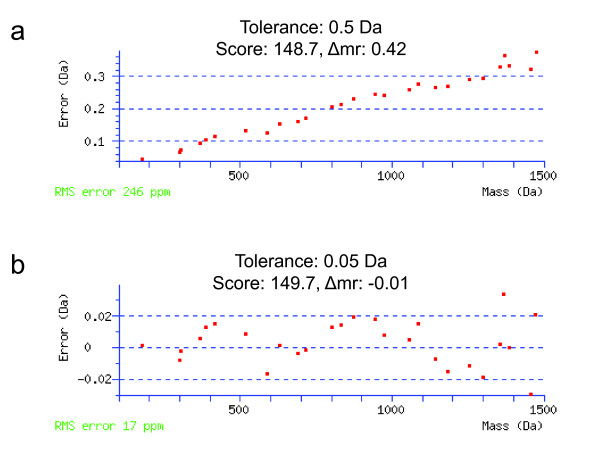
**Recalibration of Data Acquired on a Quadrupole Time-of-Flight (QTOF) Mass Spectrometer**. Fragment ion mass errors for the peptide ADISSDQIAAIGITNQR (based on Mascot assignment), derived from the protein glycerol kinase (a) before and (b) after recalibration.

In a second application, we developed a routine for recalibration of MS/MS spectra. It is widely recognized that increased mass accuracy provides for higher stringency searches and yields improved results [28]. First, a given set of MS/MS spectra are searched with mass tolerance values based on the most recent mass calibration parameters. Under these conditions we typically observe a monotonic increase in mass error as a function of fragment ion mass (Figure 10a). A high-confidence peptide is selected from the search output, and the corresponding annotated MS/MS spectrum is used to compute the slope and intercept of the linear mass error function. This equation is then used to recalibrate precursor and product ion masses via an .mz script (see Additional File 2). Finally, we re-search the newly calibrated dataset with a narrower tolerance, reducing the average mass error (Figure 10b).

## Conclusion

We recognize that some aspects of our proposal diverge from current efforts to establish community standards in proteomics. For example, the use of mzAPI within multiplierz to provide direct access to binary mass spectrometry files does not rely on XML-based surrogate files. We note however, that the two strategies are not mutually exclusive; that is, support for mzXML [9], or the recently described mzML [29] can be readily incorporated into mzAPI. Similarly, output from multiplierz can be readily formatted in pepXML [12]. In addition, recent discussions focused on data sharing in proteomics suggest that standards may evolve beyond XML-based formats [30,31]. Equally important, the emergence of translation layers such as cygwin [32] and Wine [33], continue to blur inter-platform boundaries, such that software solutions amenable to the widest audience may eclipse those based largely on platform independence. In fact, our use of Microsoft Excel as the default report output for multiplierz is one such example. Similar image-enhanced spreadsheets may be generated in open formats such as OpenOffice.org XML [34] (see Additional File 4), but our experience to date indicates that the majority of biomedical researchers still opt for commercial spreadsheet solutions, either out of familiarity or because of existing institutional support.

The multiplierz framework is accessible to a wide range of researchers, and simultaneously provides support for novel algorithm development as well as deployment of automated data pipelines. As a central point of integration for information from publically available databases and native data from proprietary instrument platforms, multiplierz offers compelling addition to the ongoing discourse aimed at identifying an effective means to enable broad access and data exchange in the proteomics community. In particular, incorporation of mzAPI into the multiplierz desktop architecture may offer a better impedance match between the rate of proprietary mass spectrometry innovation and researchers' demands for increased autonomy in their data analysis tasks.

## Availability and Requirements

• **Project name: **multiplierz

• **Project home page: **

• **Operating system(s): **Microsoft Windows

• **Programming language: **Python

• **License: **open source under LGPL

## Authors' contributions

JRP and MA designed multiplierz. JRP developed the Python code for multiplierz. MA and JRP defined mzAPI. MA and JRP developed the C++/C# and Python mzAPI code respectively. JTW and NCB developed the X!Tandem interface within multiplierz. TC integrated multiplierz within a high-throughput pipeline. SBF generated data, while MA designed and developed scripts for the label-free quantitation analysis. SBF and YZ provided the design for de-isotoping and QTOF recalibration scripts. JRP drafted the manuscript with input from all-authors. JAM conceived of the study, and participated in its design and coordination and helped to draft the manuscript. All authors read and approved the final manuscript.

## Supplementary Material

Additional file 1 **Timing Comparison Scripts**. This compressed file contains the .mz scripts, R script, Perl script and C++ program to generate timing measurements for comparing data access methods. Click here for file

Additional file 2 **.mz Scripts for Developing Custom Algorithms**. This compressed file contains all .mz scripts described in the manuscript in addition to a few other useful scripts. Descriptions.pdf contains a brief description of each script. Click here for file

Additional file 3 **Description of multiplierz Tools**. This document lists and describes the standard tools available within multiplierz. Click here for file

Additional file 5 **Example multiplierz Spreadsheet**. This is a Microsoft Excel spreadsheet report generated by multiplierz. This is the multiplierz report described in Figure 2. Hover on cell comments to view the embedded images. Click here for file

Additional file 4 **OpenOffice.org XML Spreadsheet Screenshot**. This is a screenshot describing an OpenOffice.org XML spreadsheet file that is analogous to a multiplierz spreadsheet with images embedded within cell comments. Click here for file
