# Supplementary material for: multiplierz: an extensible API based desktop environment for proteomics data analysis
Source: BMC Bioinformatics. 2009 Oct 29;10:364. doi: 10.1186/1471-2105-10-364 (PMC2774704; doi:10.1186/1471-2105-10-364)
Supplement: Additional file 2 — .mz Scripts for Developing Custom Algorithms. This compressed file contains all .mz scripts described in the manuscript in addition to a few other useful scripts. Descriptions.pdf contains a brief description of each script. [file 1471-2105-10-364-S2.zip › mzScripts/Descriptions.pdf]

Description of .mz Scripts:

.mz Script	Description
deisotope_reduce_charge.mz	Takes an MGF peak list and generates a new MGF file containing MS/MS spectra with only singly charged and monoisotopic peaks.
QSTAR_recalibration.mz	Uses a slope and intercept of the mass error linear function to adjust all precursor and product ion masses resulting in a new recalibrated MGF file.
auto_calibration.mz	Finds precursors in a MS calibration raw file corresponding to a given list of peptides, determines the mass deviation (ppm) from theoretical m/z, and generates a report with precursor scan and RIC images. auto_calibration_report.xls contains the list of calibration peptides in the "Input" worksheet and the mass deviations in the "Results" worksheet.
extract.mz	Takes a combined spreadsheet with precursor information for common peptides in k of N files and computes quantitation for each peptide using averaged information.
prot_annot.mz	Takes the output of extract.mz and generates a protein report with the median label-free quantitation ratio. "Label Free.xls" is an example of the generated report
rev_db.mz	Generates a reverse fasta database given an input fasta file
FDR.mz	Calculates the false discovery rate given forward and reverse database search results
ric_draw_app.mz	Demonstrates a simple GUI application that draws an RIC at specified time and mz windows
entrez_aka.mz	Downloads all synonyms of an Entrez gene name and appends this information to a multiplierz GenBank report spreadsheet
