# Supplementary material for: multiplierz: an extensible API based desktop environment for proteomics data analysis
Source: BMC Bioinformatics. 2009 Oct 29;10:364. doi: 10.1186/1471-2105-10-364 (PMC2774704; doi:10.1186/1471-2105-10-364)
Supplement: Additional file 3 — Description of multiplierz Tools. This document lists and describes the standard tools available within multiplierz. [file 1471-2105-10-364-S3.pdf]

Description of multiplierz Tools:

Tool	Description
Download Mascot	Automatically download and generate Mascot reports as multiplierz spreadsheets
Multifile Detect	Detect number of occurences of combinations of protein, peptide sequence, variable modifications, and charge sate across multiple multiplierz spreadsheets
Filter Multifiles	Filter multiplierz spreadsheets for a specified list of proteins or peptides
Download GenBank	Generate genbank report by automatically downloading and parsing genbank files for proteins in a multiplierz spreadsheet
Fragmenter	Generate in-silico peptide fragmentation pattern for commonly occuring ions
Digester	Generate a list of enzymatic digestions using a variety of enzymes for given protein(s)
Retrieve Peaks	Extract precursor, chromatographic, and ms/ms information from mass spectrometry raw peak files using mzAPI
Mascot Web Extract	Download information such as protein coverage and fragment identification on MS/MS images stored in Mascot web reports
Formatter	Convert Mascot CSV and Protein Prophet Peptide Summary files to multiplierz spreadsheets
Console	Interactively script the multiplierz libraries and environment for mass informatics exploration
Peak Viewer	Interactive visual environment for in-depth interrogation of mass spectra
Combined Peaks	Retrieve Peaks for merged searches or combined search results
Full Report Wizard	Download Mascot results, Mascot web information, Genbank information, and peak information through one tool
