# Supplementary figures and images for: multiplierz: an extensible API based desktop environment for proteomics data analysis
Source: BMC Bioinformatics. 2009 Oct 29;10:364. doi: 10.1186/1471-2105-10-364 (PMC2774704; doi:10.1186/1471-2105-10-364)

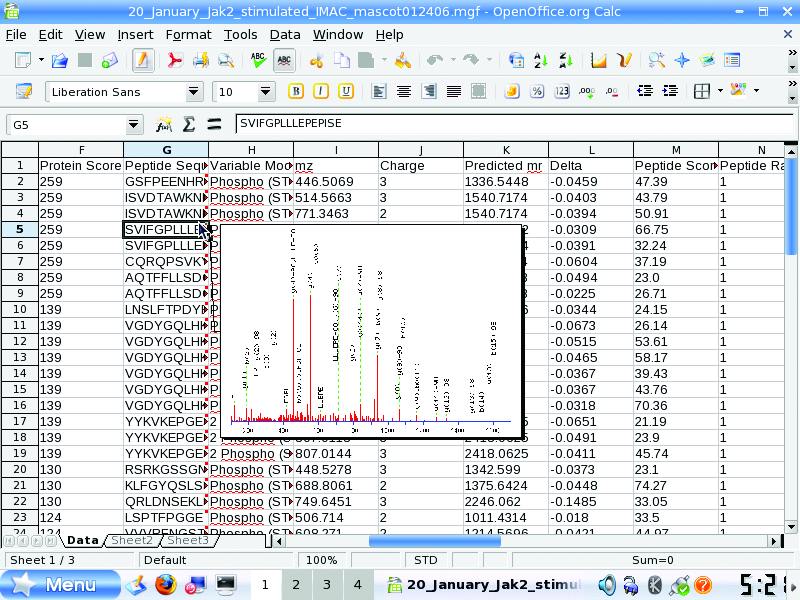

Supplement: Additional file 4 — OpenOffice.org XML Spreadsheet Screenshot. This is a screenshot describing an OpenOffice.org XML spreadsheet file that is analogous to a multiplierz spreadsheet with images embedded within cell comments. [file 1471-2105-10-364-S4.tiff]
